# Extract, model, refine: improved modelling of program verification tools through data enrichment

**DOI:** 10.1007/s10270-024-01232-7

**Published:** 2025-01-08

**Authors:** Sophie Lathouwers, Yujie Liu, Vadim Zaytsev

**Affiliations:** Formal Methods and Tools, University of Twente, Enschede, The Netherlands

**Keywords:** Program verification, Megamodelling, Data enrichment, Data extraction

## Abstract

In software engineering, models are used for many different things. In this paper, we focus on program verification, where we use models to reason about the correctness of systems. There are many different types of program verification techniques which provide different correctness guarantees. We investigate the domain of program verification tools and present a concise megamodel to distinguish these tools. We also present a data set of 400+ program verification tools. This data set includes the category of verification tool according to our megamodel, practical information such as input/output format, repository links and more. The practical information, such as last commit date, is kept up to date through the use of APIs. Moreover, part of the data extraction has been automated to make it easier to expand the data set. The categorisation enables software engineers to find suitable tools, investigate alternatives and compare tools. We also identify trends for each level in our megamodel. Our data set, publicly available at https://doi.org/10.4121/20347950, can be used by software engineers to enter the world of program verification and find a verification tool based on their requirements. This paper is an extended version of https://doi.org/10.1145/3550355.3552426.

## Introduction

Program verification (PV) is a field that has always enjoyed very high expectations, and suffered from them as well. Its objectives are mostly to provide ways to prove that a system satisfies certain requirements. The underlying *techniques* are typically based on rigorous mathematical reasoning or an exhaustive analysis of the state space, thereby giving software engineers stronger guarantees than testing. It is often accepted that to use program verification (or formal methods in general), one needs to specify their system in a formal notation and thus have considerable formal background to do it in a correct and useful way [22].

To simplify, for the rest of the paper we use the established team “program verification” to mean verification (conformance evaluation) of programs (executable models). Hence, it covers generative techniques, testing, model checking, theorem proving, etc, of source code, automata, Petri nets, transition systems, etc.

Adopting verification *tools* has shown to present not only technical challenges, but also organisational, social and managerial ones [28], similar to challenges faced by advanced model-driven engineering tools [49]. PV tools are particularly difficult, because even demonstrating potential benefits of their use is highly nontrivial and relies on users having very specific knowledge of the underlying techniques. For the tool developers, the tools themselves often serve as a means to an end, as an opportunity to demonstrate the extent of applicability of their techniques, to exemplify the problems that could possibly be tackled, and to enter an existing subdomain. Some subdomains are accompanied by sets of mature benchmarks which make comparing techniques by comparing tools a very attractive and attainable goal. Examples include programming language theory [8], software verification [16] and quantitative verification [44]. Admittedly, many tools stay in a prototype phase, and being actively developed only till a certain point: until the tool can handle the minimal set of benchmarks, or until the deadline for submitting the paper explaining the underlying techniques, or until graduating from a PhD project.

Besides techniques and tools, there are multiple *sources* of information to consider. Papers themselves are an obvious source, well-archived on publishers’ websites, but requiring high qualifications to be considered readable and understandable. They are also hard-dated, meaning that an average good paper contains detailed comparison of the proposed tool with its existing counterparts, but no comparison or relation to counterparts that were created after the publication. The papers often refer to product or project pages, which are prone not only to being outdated for reasons mentioned above, but also to being removed due to the jobhopping nature of the academic world: when the principal investigator finishes the project and moves to another institution, it is not guaranteed that the project page will be preserved by their original employer. If available, such websites are also wildly varying in the nature of their content: some literally repeat the contents of the papers, while others complement it with valuable information, illustrations, and links.

Another extremely valuable source of information — primarily about the tools and not always about the techniques — is the code repositories. It has become fairly commonplace in recent years to either release the tools for (limited) public use to enable empirical replicability, or expose the entire development history in a form of versioned codebase (typically through git, occasionally hg or svn). There are at least three benefits of repositories: (1) the artefacts become much more tangible, and only require several natural steps (like cloning the repository) to set themselves up on the user’s computer instead of extracting them from the paper text; (2) the version history is a technically substantiated claim to the amount of work and to its authorship; and (3) linking tools to one another by shared contributors plays the same social role as linking papers by shared coauthors.

To summarise the problems:

- existing *techniques* are hard to understand and assess their applicability without very deep specific knowledge;
- *tools* are hard to classify conceptually and appropriately relate to *techniques*;
- information *sources* are dispersed, partly unavailable and partly unreliable.

With the vision to open up the arsenal of PV tools and techniques to a broader public of software modellers and, even broader, software engineers, we have developed a megamodel of program verification tools. The megamodel can help answering questions like “what am I expected to provide as input to use tool X?” or “what other tools exist for the same problem domain as tool X?”, or even “when was the last time the code of tool X was updated?”. We will elaborate on the megamodel and its **PV0**–**PV6** levels in Sect. 3.

With this megamodel, we show that there are many different types of PV tools, and those types can be grouped in categories that form a hierarchy. Thus, if a tool from one category comes fundamentally short to solve the end-user’s problems, it can be considered to seek alternatives in a broader category. To concretise the megamodel, we complete it with a data set into which we have collected information about 450+ PV tools, frameworks and languages, published recently at two top conferences in the PV domain known for their tool paper hospitality: CAV and TACAS. The data set is available publicly on GitHub, with a reader-friendly interconnected hypertextual frontend at https://slebok.github.io/proverb.

We strongly believe that making the data set freely accessible for exploration, makes it an attractive starting point for software engineers to traverse the domain of program verification. Section 4 contains more information about the data set, as well as our methods of gathering the data, categorising and enhancing it.

In Sect. 5, we report some preliminary lessons we have learned ourselves by looking at the collected information, level by level, and analysing some of its trends. The fact that our megamodel splits the PV domain into distinct groups recognisable from prior research, is considered here as a form of evaluation and evidence that the megamodel is viable and useful.

This paper is an extended version of the conference publication by [58]. Besides going into more details for the lack of space constraints and in order to make this paper more self-contained (with respect to its own website and repository), the additional contributions here are:

- Linking this line of research to the current research trends on artefacts and artefact evaluations (Sect. 2).
- Automating further expansion of the data set by using heuristics to identify tools in new publications (Sect. 4.1.2), since providing tool support in updating the data set is instrumental for its continuous maintenance and growth.
- Automating information extraction from conference proceedings (Sect. 4.1.3), significantly lowering the barrier to add new entries.
- Refining and enriching the data set “horizontally” by adding more information, more precise information or more up to date information with the help of several APIs (Sect. 4.2). For instance, the date of the last update does not have to be manually checked but is inferred from the git history of the tool repository.
- Providing a more conceptual/clean approach to model each tool based on an ontology (Sect. 4.2.1) instead of an ad hoc template — cf. [58, section 3.1.3].
- Using these methods for extending the data set into several dimensions (Sect. 4.2) and reporting statistics on that (Sect. 4.3).
- Planning an extensive research roadmap for the future of this project (Sect. 6).

## Related work

When it comes to related work, we refer the attention of the readers to in-depth overviews of problems in adoption of program verification, formal methods, model-driven engineering and domain-specific languages, which we summarise below.

In 2005, Bodeveix, Filali, Lawall, and Muller [19] provided a side-by-side comparison of two solutions to the same case study problem (kernel-level process scheduling): one done with software modelling (in particular, a domain-specific language), and the other with formal methods, with a conclusion that a formal specification provides at least as good of a model as a DSL one. The modern view on this matter is to combine the power of two sides of this debate, and link program verification to formal DSL models [5]. This can be done either way: by augmenting the existing system with an internal DSL and using information encoded there for verification purposes [12] or by generating formally correct DSL code [68].

Broadfoot and Broadfoot [22] argued that in the domain of embedded software, there are many systems which are both business-critical and untestable with conventional means. Their concluding advice was to rely on provably correct designs which are transformed into code by using only tools that are provably correct. Woodcock, Larsen, Bicarregui, and Fitzgerald [91] provide what seems to be the most extensive survey on formal methods, their prevalence in academic literature and in industrial experience. They also listed a few of the most popular tools by name, without introducing any exhaustive coverage criteria like we will do in Sect. 4.

Bucchiarone et al. [23] stressed the important of informal modelling as a way to attract new users to MDE. In this case, informal modelling does not mean the lack of formal methods: it designates the ease of use for novice modellers and the ability of the metamodel to express incomplete and uncertain information. Under the hood such products can still rely on highly complex science in the same way an internet search query relies on MapReduce and data synchronisation with a mobile device relies on lenses.

Davis et al. [28] conducted a large study of 31 experts from 9 companies, and identified that the top three barriers for adopting formal methods in the industry are education, tools quality and personnel changes, while the top three improvements were education, tool integration, and creating and disseminating evidence of the benefits of formal analysis. Tomassetti and Zaytsev [83] combined personal experiences of two industrial experts in the field of domain-specific languages and summarised the real advantages of using DSLs next to their adoption problems of different nature.

Klösch and Eixelsberger [52] provided a fairly extensive yet concise list of explicit and factual challenges that the software industry faced when solving the Y2K problem as well as conversion from local currencies to Euro. Their structured list was also broad in nature and covered many aspects from formal compliance validation to organisational workarounds. More than two decades later, Nurwidyantoro et al. [66, 67] as well as Whittle, Ferrario, Simm, and Hussain [86] argued that human values such as responsibility, transparency, creativity and equality represent a substantial fraction of software engineering challenges and difficulties, yet are heavily underrepresented in methodological research.

If we were to summarise all these extensive studies on relatively well-known problems, we would have to admit that most of them are related to tools, their prototype-level quality, notorious unavailability, lack of support and sufficiently reliable documentation, etc.

Over the years, many ontologies, taxonomies and surveys have been published about program verification. However, these works tend to target either a specific subset of program verification, e.g. runtime verification [36], high-level synthesis [61] or a specific domain, e.g. vehicular domain [78], smart contracts [45], railway system design [37], programmable logic controllers [40]. Unlike these works, we do not focus on a subset of PV, but aim to deliver both a megamodel to explain tools, as well as an easily accessible (and extensible!) repository with a large data set of classified tools.

The work that is closest to ours is a report by Punnoose, Armstrong, Wong, and Jackson [72] that presents several verification techniques in detail. It covers a broad variety of techniques including model checking, verification condition generation and correct-by-construction design. However, they only mention a few tools per technique, whereas we consider all PV tools that we could identify in publications. Nonetheless, this report can also be a nice starting point for engineers.

When it comes to repositories, we also do not claim outright novelty. Over the years, several projects have tried to achieve more or less similar goals. For instance, the Verified Software Repository [18] was intended to become a collection of tools, verified programs, benchmarks and results. Unfortunately, it was last updated in 2009. Schlick et al. [77] have also proposed to set up a repository to make formal methods more accessible to users in the industry. A large part of their work focuses on the obstacles that limit the adoption of formal methods in industry. Some of these obstacles include the maturity of tools, difficulties in finding the right methods and lack of ways to easily compare different tools. This is in line with our experience and the research mentioned in Sect. 1. Like us, Schlick et al. [77] believe that the awareness and comparability between formal methods should be improved. To achieve this, Schlick et al. [77] use notable success stories to identify key information that should be included in a formal methods repository. They then present a vision of a repository and why it would be beneficial. They give a repository structure that includes (1) experiment data, (2) applications, (3) problem descriptions, (4) formalisations, (5) usage patterns, and (6) tools. However, to the best of our knowledge, this repository has not yet been instantiated, and remained a dream. One could consider our data set as a first instantiation of the “Tool" part of their proposed repository structure. In the future it could be combined with other data to form a complete repository as proposed by Schlick et al. [77], including data such as experiments and applications.

An important aspect for tools in our data set is their availability and reusability. There have been several efforts to evaluate the reusability of artefacts. Baldassarre, Ernst, Hermann, Menzies, and Yedida [10] present the Department of Reuse that records the reuse of research, including 8 different types of reuse such as tool reuse and data set reuse. Both Winter et al. [89] and Hermann, Winter, and Siegmund [47] have gathered data to evaluate whether artefact evaluations were as effective as hoped. Where Winter et al. [89] focus on the availability of artefacts, documentation practices, hosting platform and citation count, Hermann, Winter, and Siegmund [47] take a different approach and focus on the community’s expectations for such artefacts. We consider all these contributions to be complementary to our research as they could provide information about the (re)usability of tools in our data set. While we are interested in such data, our interest is in whether tools are usable in practice as opposed to its implications for the academic sphere in terms of the effectiveness of artefact evaluations or its relation to citation counts which is explored in the previously mentioned works.

Other directions for future work and consequences of this project, are considered in Sect. 6.

## The megamodel of PV-levels

**Fig. 1:**
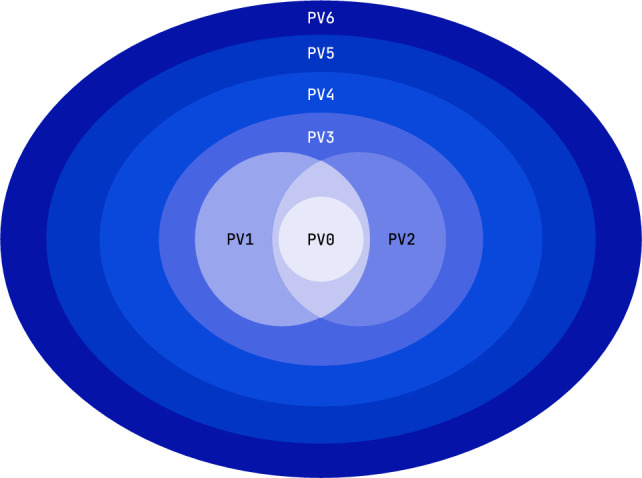
Each ellipse indicates the potential correctness guarantees that can be acquired by using a tool of that level. PV0 tools give the least guarantees of correctness, whereas PV6 tools allow the user to work towards maximum correctness guarantees. Note that these indicate the potential of each level; a tool may only support a little piece of a level

In this section, we introduce the seven levels of our megamodel: **PV0**, **PV1**, **PV2**, **PV3**, **PV4**, **PV5** and **PV6** (see Fig. 1).

Intuitively, higher levels give the user more correctness guarantees, though typically at the cost of more user effort, and lower level tools are usually less strict and thus do not require as much PV expertise to be effectively applicable.

Since the ultimate goal of PV is to prove correctness of the artefact in some form and within some domain, we will use the classic division of roles in a correctness proof. It was originally introduced by Goldwasser, Micali, and Rackoff [41], we use the more widespread modern terminology here [88]: 
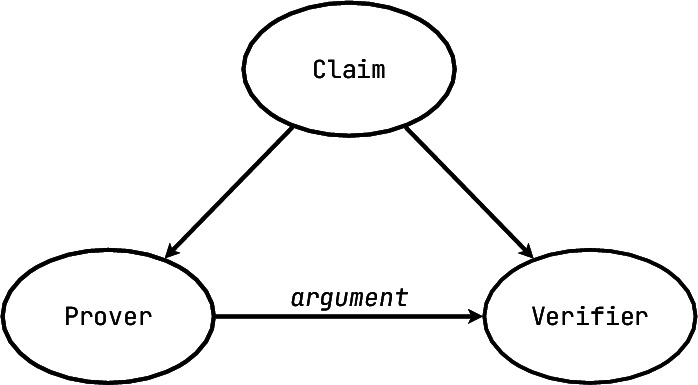
 In short, there exists a **claim** of some sort (e.g. “$x \in S$” or “the program has no memory leaks” or “all models are wrong”), which is provided to both the prover and the verifier. The **prover** is very clever and can perform sophisticated manipulations and computations. Its goal is to produce arguments supporting the claim, but the prover can also be biased and prone to producing false positive arguments. The **verifier** has some way of checking the **arguments** and, depending on its verdict, declaring the claim accepted or rejected. We will be illustrating each of the PV tool levels with explanations, examples and also differences on this simple scheme. In the subsequent diagrams we will also use green colour to highlight the main contributing elements that make someone decide to use a tool of this particular level. 
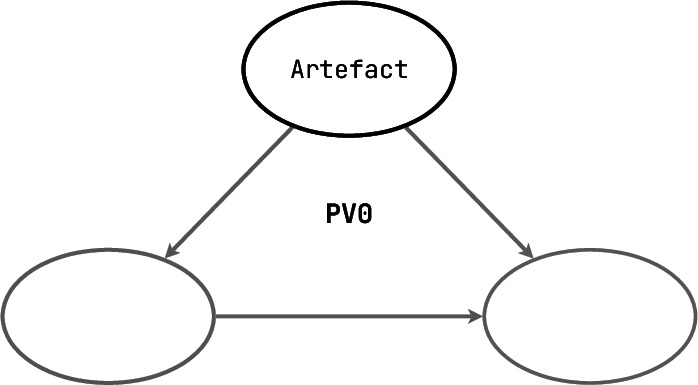
 [**PV0**] Software engineers always work with abstractions and models of reality. Once a software entity satisfies the three properties of the modelling theory [81], it can be seen as a model. These three are the mapping property (elements of the model represent some elements of the real entity being modelled), the reduction property (only the most important aspects of the real entity are being modelled and others are being abstracted from), and the pragmatic property (the model has a purpose). **Formal models** are a subset of such models, which are clean and well-formed, and often built with the use of some existing mathematical theories. For example, formal models often cover domain-specific variations of automata. In **PV0**, such a formal model may exist but it is often implicit and is used neither to obtain nor to verify any correctness guarantees. 
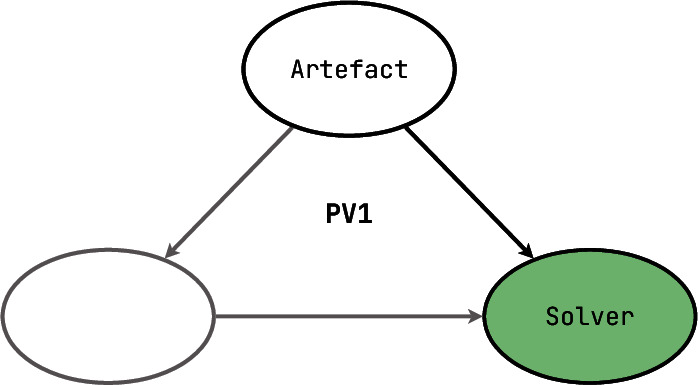
 [**PV1**] Once a formal model can be operated on by a software system, it can also be automatically checked for internal consistency and well-formedness, by a **model solver**. For instance, if the underlying theory states that a model of a process is some specific automaton with one starting state and one or more final states, and all transitions labelled with unique names, then a solver can check that all these properties indeed hold. The more complex the model, the more difficult it could be to make such a solver for it: for example, uniqueness is relatively easy to check on strings (which we assume for transition labels in our previous example), but it is noticeably harder to define and enforce even on database records, where single columns (such as “first name”) often contain non-unique values, and combinations are often unreliable due to incompleteness and subtle tolerable inconsistencies (such as a phone number mismatch). From the correctness perspective, a **PV1** tool plays a role of a verifier, and a prover does not exist since the verifier does not need any arguments on top of the ability to observe the given model. 
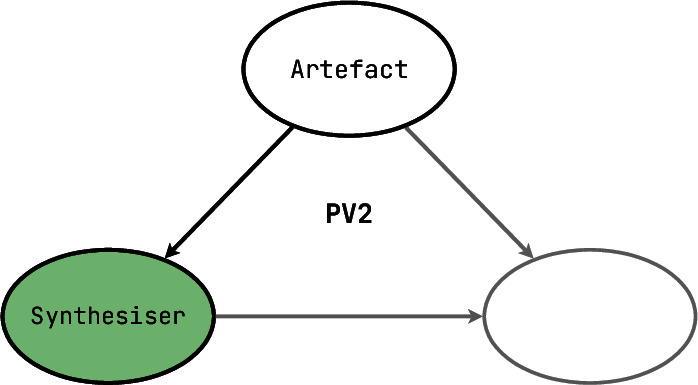


[**PV2**] The opposite situation is also commonplace: if a user makes a model of their wishes, often taking a form of an almost-consistent artefact with holes to be filled, then one can build a tool to fill in those gaps and infer them from the context. Sources of information can be different, ranging from domain common sense (for example, we obviously want our parallel programs to not get stuck waiting for one another’s resources) to constraints and instructions explicitly specified by the user. In a sense, if we want to consider the Eclipse Modelling Framework as a proof system, it would fall into this category because it can produce the textual code of classes that conform to the inheritance structure and the interfaces specified in the class model. In PV, such programs are often said to solve problems of **synthesis** and **repair**, and use generative techniques to create test data, repair known categories of defects, implement queries, generate neural networks fitting for a particular grid, or just to configure a universal algorithm with automatically obtained balanced values. **PV2** tools help users to create software artefacts — either by generating them from scratch or by providing significant assistance in the incremental process of creating them semi-automatically. If the output of a **PV2** tool is expected to be processed by another automated component, then the tool belongs on a higher PV-level. 
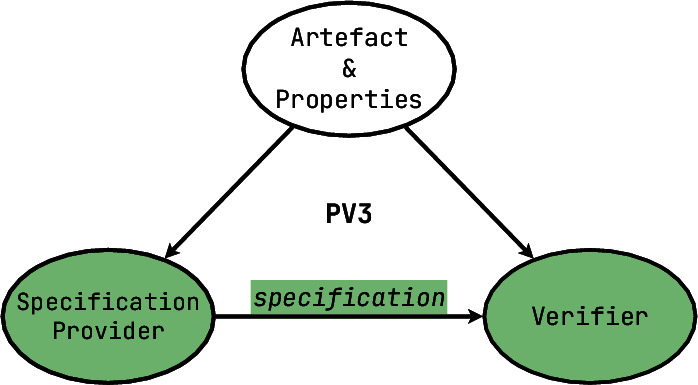
 [**PV3**] Combining the two components into a] symmetric setup (cf. Fig. 1), in the simplest case we get a situation when a user explicitly states what properties of a formal model they wish to have (beyond well-formedness), and there is an automated **property checker**, conceptually decomposable into two parts: a prover that turns each property into a convincing argument and a verifier which validates the convincing power of such arguments. A typical example of a property checker allows the developers to add assertions to their code, specifying preconditions, postconditions and invariants around a code fragment, thus allowing additional formal ways to validate its correctness. These assertions do not have to be deployed to the end-user, but serve as a powerful tool for the developer to verify the product beforehand. Some checkers have a very extensive formal language to write properties in, usually a variant of some special kind of logic, e.g. temporal logic [57, 71]. 
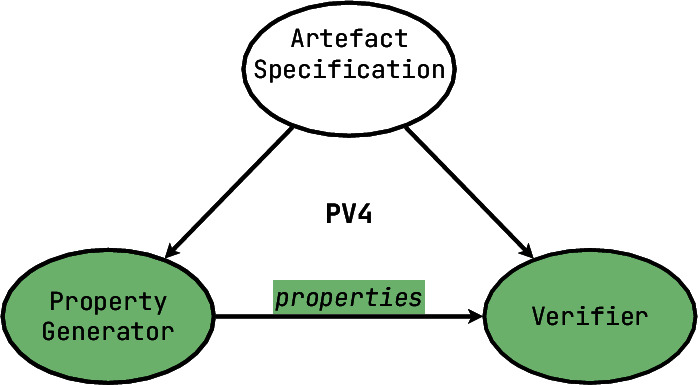
 [**PV4**] On the previous level, the burden to create verifiable properties, was on the end-user of a tool: assertions had to be explicitly written, and invariants had to be provided. However, in some cases it is possible to automate the creation of desired properties as well as their verification — since such techniques require an extensive specification of the desired behaviour, and often focus on only one paradigm, we call them **monoverifiers**. They are very useful tools in debugging, because if used correctly, they can significantly lower the chances of having a particular category of defects, sometimes up to eliminating the very possibility of such a defect ever occurring. For instance, think of a parallel system being checked for deadlocks or a garbage collector checked for the lack of memory leaks. Essentially, monoverifiers verify that the supplied formal model corresponds to the expectations of their own built-in specification.

Some monoverifiers offer a choice of checking one or more of a larger set of correctness specifications, in which case we still classify them to belong to **PV4**, even though the *mono-* prefix no longer fits — as long as the end-user has no direct control over the specifications themselves. Also most monoverifiers embrace the fact that their generated properties cannot always cover the end-user’s needs, and allow for direct manual specification of additional properties — which allows us to claim that **PV4** functionalities are a strict superset of **PV3** functionalities. 
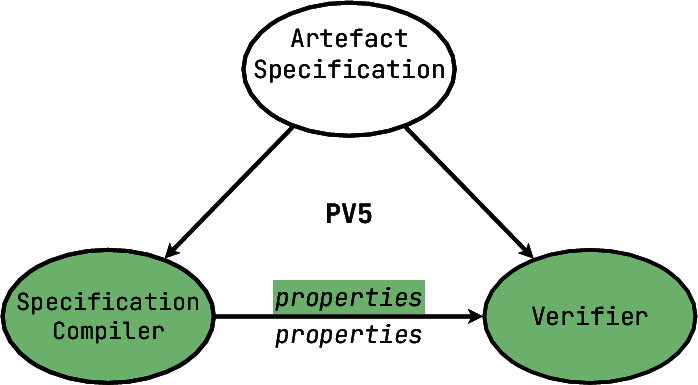


[**PV5**] When the tool users have an opportunity or obligation not only to specify which properties of the system to check for or how to infer them, but also to build their own specifications, we get to **specification compilers**. Such compilers usually have a language used to write specifications in, sometimes based on a domain-specific notation, and support this entire language by compiling its instances in some automated way to verify their correctness and compatibility. With those, you can build your own specifications of memory management strategies, your own communication protocols, and so forth. To continue with examples from the previous paragraph: when a **PV4** tool could check for deadlock freedom, a **PV5** tool would require a formal specification of the concurrency model, accompanied with a definition of what constitutes a deadlock state. Obviously, some **PV4** tools are built on top of **PV5** frameworks by essentially supplying a useful singular model. 
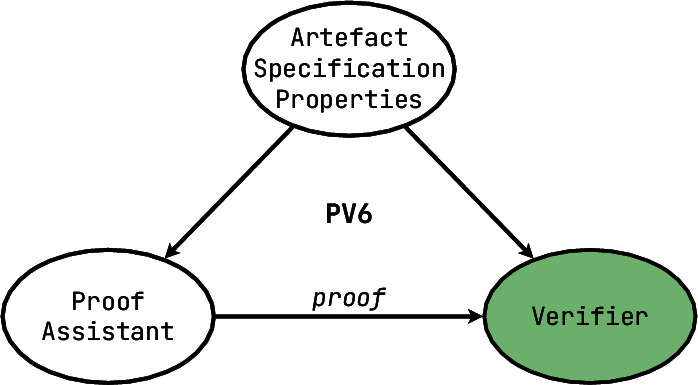

**Fig. 2:**
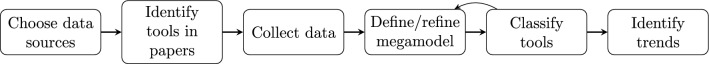
An overview of the different steps that have been undertaken to set up the data set of program verification tools

[**PV6**] If your program verification tool can not only handle different specifications, but also infer correctness of the proof of the needed property, then it belongs among the **proof assistants**. This category is the most powerful one that we have encountered, which means both that it is the hardest and most demanding to use, as well as capable of producing the strongest guarantees. However, as one can see from the diagram we provided, it bears some similarities with the **PV1** level, since there is very limited automation and tool support in composing the arguments for correctness. The proof needs to be written by the end-user, and the tool can only offer some assistance in verifying that the proof is indeed correct. Some of **PV6** tools can *feel* to their users as if they also help them to compose the steps of the proof, but under closer inspection this help comes from the tool knowing which proof step would succeed in reaching a user-stated goal, and not from the tool relying on some generative algorithms. Within the claim/prover/verifier paradigm, proof assistants offer powerful techniques on behalf of the verifier and not the other two components.

## Data set of verification tools

To help users find a suitable PV tool, we have prepared a data set of tools categorised according to the megamodel we have just explained in Sect. 3. This makes it easier to discover which tools are available, to find tools using similar techniques, as well as to find tools that target similar domains and problems. The data set, called **ProVerB**, is available^1^ at https://slebok.github.io/proverb/. Each tool has its own file which contains all of its data and metadata in Markdown format.

The remainder of this section explains how the initial data set has been created (Sect. 4.1), how we use open APIs to enrich our data set (Sect. 4.2) and presents some statistics about the data that was gathered (Sect. 4.3).

### Methodology

A methodological overview can be seen in Fig. 2. Below we will describe each step of our research method in detail.

#### Choose data sources

To find PV tools to include in the data set, we looked into two popular conferences about verification of systems. Namely, the International Conference on Computer Aided Verification (CAV) and the International Conference on Tools and Algorithms for the Construction and Analysis of Systems (TACAS). In the CORE conference ranking, they are classified as having respective ranks of A*^2^ and A.^3^ We have decided to use papers from these conferences since both accept and welcome papers about tools. Therefore, we could expect a relatively high percentage of tool papers. Moreover, CAV and TACAS started with artefact evaluations in 2015 and 2018 respectively, therefore we expect that many of the tools presented here will also be available. We have looked at tool papers from TACAS 2016–2021 and all papers from CAV 2017–2022. We are still in the process of adding the TACAS 2022 papers. We chose to use these recent years as this makes it more probable that tools are still findable and possibly maintained, yet still limited ourselves to at least 5 years of each conference in order to gather a substantial amount of data.

This step resulted in 519 papers: 406 from CAV and 113 from TACAS.

#### Identify tools in papers

Next, we needed to identify the tools that were presented in each paper. To help with this process, we have developed a script that automatically identifies paper titles in the proceedings. Based on heuristics we try to identify the name of a tool. Specifically, one can often find titles of the form “Tool name: subtitle with explanation". Whenever a paper’s title matches this heuristic pattern, we automatically extract the tool’s name. For papers that do not adhere to this heuristic it is still required to read the paper to identify tools.

For each paper we checked whether it contained a reference to a tool. If so, then we would tag this as one of the following claims:

- **Presents**: the paper introduces a new tool;
- **Extends**: an existing tool gains new functionality in the paper;
- **Expands**: the paper uses an existing tool as a basis for building another tool;
- **Uses**: the paper uses an existing tool for a case study or to check the correctness of an approach.

We only included tools that provided some form of correctness guarantees, to avoid including too many entries in ProVerB. This still left us with some entries that were later reclassified as not belonging to the PV domain (usually from misinterpretations of claims “we use library X”).

#### Collect data

As a format for storing entries in the data set, we have chosen Markdown. This provided the lowest entrance barrier and maintenance cost, still combined with the opportunity to add structure to the data (in our case, in the form of ####-level sections). By choosing this format, we also hope to make it easy for other people to contribute to the data set in the future, since GitHub, our hosting platform for the data, even provides inline editing functionality for Markdown pages.

After some pilot classifications we set up a generic template for tool pages, which has proven to be quite resilient, and after the first couple of sprints it stayed stable and unchanged till the current moment. This template included a section for all the information that we were interested in for a tool, namely:

- Name of the program verification tool;
- Domain or application field;
- The type of the tool as self-identified by authors;
- Input that is required from the user;
- Input format;
- Output that is produced;
- Internal working of the tools, such as which tools it uses as a backend;
- External relations to other tools, such as those that were compared to this tool in the paper;
- Links to project pages, repositories and related papers;
- Dates when the tool and its documentation were last updated;
- Reason why the tool was added to the data set.

Aside from the information mentioned above, we have also started adding tags as textual annotations. Tags are used to indicate whether a tool targets a specific language, domain, technique, etc. This should make it easier for users to find suitable tools. For example, there is a tag for tools that target C programs, a tag for neural networks, a tag for hardware verification, and so forth.

We start by creating a new page based on the same template for all tools. After the page has been created, we needed to collect additional information from the corresponding paper as well as the code base and project website if these were available. Some sections were left empty if the data was not available (e.g. the last modification data for tools without a repository). If at least two tools referred to another tool, e.g. because it was used as a back end or as a framework, then this tool was added as well and received its own entry.

Some tools that we encountered were developed as prototypes, up to the point that these did not have a name at all, nor a link to an implementation. We decided to exclude such tools as these tools were likely not developed and maintained for professional use. However, some tools included an artefact, which was mostly still reliably available, so we included this link in the entry whenever it existed.

The data set also includes pages about several specification formats. A page for a format was created if the format was not tool-specific, if it was used by more tools than one, or if it was for some other reason conceptually separate from the tool.

#### Define megamodel

When the tool pages had been written, we contemplated the initial setup of the megamodel based on similarities between tools. The first version was already based on the input that the user has to provide, ranging from the no input at all (besides the already existing software artefact), to assertions, properties, specifications, theories and proofs. Several refinement iterations later, based also on consulting the already available domain knowledge [41, 88], we have arrived at the version presented previously in Sect. 3.

#### Classify tools

Having designed the initial megamodel, we started the process of classifying all the tools. Based on the tools’ semi-structured description (cf. Sect. 4.1.3), we have assigned each to a PV-level. While doing that, we have also consistently provided a short description motivating this classification by explaining what the tool does. In that way, a tool with a description “verifies properties of a user-specified memory model” was clearly placeable at **PV5**, and the one with “checks user-specified properties and memory safety of C programs” was easily marked as deserving **PV4**. To prevent misclassifications and improve inter-coder reliability, the authors actively double checked each other’s verdicts and had extensive discussions about arguable conclusions. In such discussions, the classifying coauthor would usually apply the definition of the chosen PV-level to argue that the tool conforms to it, to be challenged by another coauthor with an alternative application of a different PV-level. In case of conflicts, the original tool describing paper was consulted for more detailed information sufficient to finalise the classification.

Aside from the **PV0**–**PV6** levels, there are two other categories: “no PV” for false positives and “frameworks” for a possible level mixture. We used “no PV” to explicitly exclude entries that ended up, after close consideration, not performing any PV-related tasks. Such entries were mostly about specification formats, but also about IDE plugins, unrelated programming languages, libraries not performing any PV tasks, etc. We felt that something like an alternative user front end or a linear programming library do not belong to **PV0** either. “Frameworks” was used to classify collections of tools: in many cases it was possible to determine the primary objective of the collection and assign a framework to a proper level as well, but in other cases such an assignment has not been deemed sensible. For example, “Alloy” is used to refer to the Alloy Analyser (which has its own entry on **PV3**), or to the input level of the Alloy Analyser, or to the entire ecosystem of Alloy models and their verifiers, — and is not consistently PV-classifiable without explicit disambiguation.

#### Identify trends

Finally, after we classified all the tools, we could start to identify trends in each level of the megamodel. These trends could be identified based on the short descriptions that were written in the previous step, and require only occasional lightweight double checking with the full data entry or the text of the underlying paper. We will discuss these trends in more detail in Sect. 5.

### Data enrichment

The previous section has described how we set up the initial data set. This section will describe how we have gathered additional data by combining the initial semi-structured data set with data from third-party open APIs such as GitHub and Springer. For this process two main requirements were taken into account namely:

- The structure of the data set should be preserved.
- The process should be automated to minimise manual effort required for maintenance

The data set can be enriched in three independent dimensions:

1. Adding new entries for tools originally not present in the data set (e.g. from newly published papers).
2. Adding new information about tools which are already in the data set (e.g. the time of the last update in the code repository).
3. Improving the information already present about the tools (e.g. adding the titles of referenced papers).

**Fig. 3:**
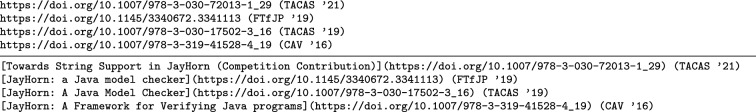
Improvements in the “List of related papers” section for JayHorn: the before and after versions above and under the line. Note how manually added information (conference names with preferred abbreviations) is still preserved after refinement

For the (1) dimension, we have only reached partial automation of the first step described in Sect. 4.1.3. We use a script to automatically extract some information from the conference proceedings using text pattern matching on the PDFs. Specifically, we automatically try to identify the tool’s name (as mentioned before in Sect. 4.1.2), links to additional resources and keywords. The tool’s name and links are automatically added to the appropriate sections in the template. The keywords are used as an initial set of tags.

For the (2) dimension, one of the enhancements we found useful is to rely on GitHub API instead of on manual investigation, to determine the time when the repository (either determined by the PDF parser or added manually) was last updated. This is not only fast, but also a much more reliable process, which can be repeated as often as we would like (which makes sense for tools that are actively being developed or maintained).

This brings us to the (3) dimension, which seems cosmetic, but improves the experience of using ProVerB nevertheless. For instance, originally, the data set only contained bare links (URIs) for papers and repositories due to which the users could not see, which paper they would be redirected to. As a result, users often needed to click and browse several links in order to find information that they needed, especially for tools described in several papers. To make this process more pleasant, we use API to automatically gather data about what the links point to. This way, we can automatically retrieve the latest commit date for repositories due to which users no longer need to click on the repository link to see whether it is still maintained, but also to replace faceless DOI URIs with hyperlinked paper titles with Springer API.

To see a concrete example, let us focus on JayHorn, a tool we randomly selected to serve as another example in Table 2. Originally, its “Last commit date” section contained the string “27 May 2021”, which was added manually by one of the authors of this paper who classified it first. After expanding in the (3) dimension, it contained more concretely two reference points: “27 May 2021 (default branch)” and “14 Dec 2021 (last activity)”, which was updated on 12 November 2023 when Philipp Rümmer, one of the contributors, merged pull request #161. For the same entry, the section about related papers has changed as depicted in Fig. 3.

#### Workflow

To set up the automatic data enrichment, we first designed an ontology for ProVerB which is closely related to the template described in Sect. 4.1.3.

**Fig. 4:**
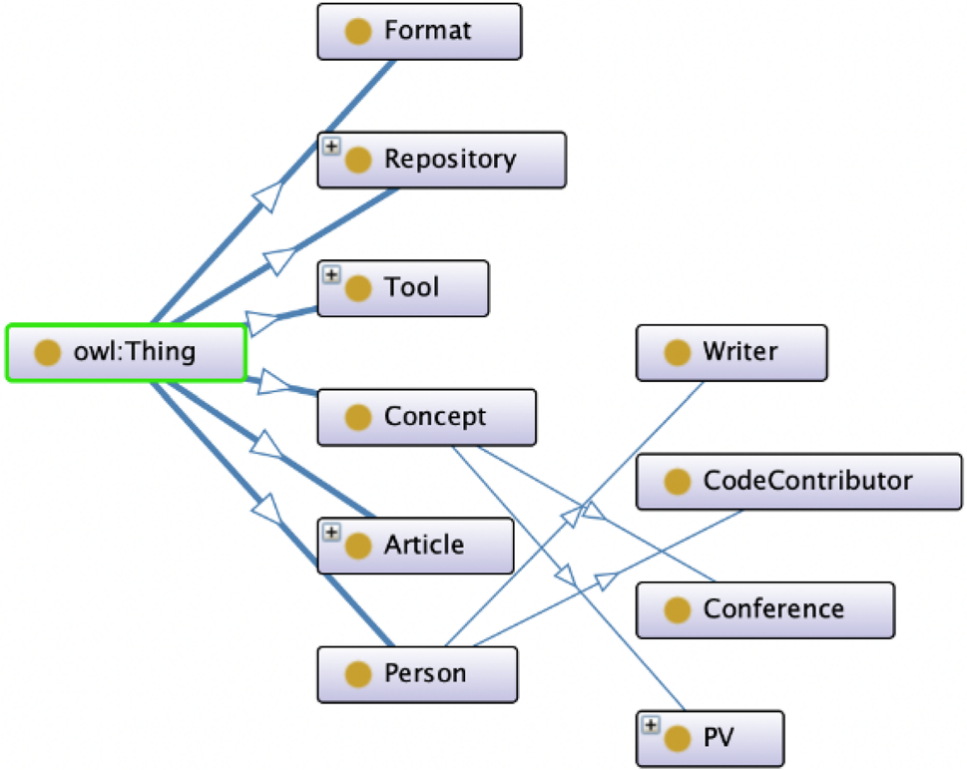
ProVerB ontology

The current version of the ProVerB ontology includes 11 classes (see Fig. 4). The meaning of each class as well as an example can be found in Table 1, with a concrete example in Table 2. The classes that we are currently using for data enrichment include Repository, Article, CodeContributor and Writer. Links to repositories and articles from the original data set are used to instantiate the Repository and Article classes. These links are then used to gather additional information, such as authors and code contributors, through open APIs. This additional information can be used to instantiate the classes such as CodeContributor and Writer.

There are some classes in the ontology that are currently unused but have been added for future study. For example, the Format class has been added to explore possible input–output relationships between tools.

**Fig. 5:**
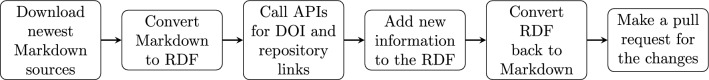
An overview of the different steps that are undertaken each time the data set is enriched

With the ontology designed, we could now use this to enrich our data set. The steps of this process have been illustrated in Fig. 5.

**Table 1:** Overview of the classes in the ProVerB ontology with an explanation of their meaning

Class	Meaning
Tool	A program verification tool in the ProVerB data set
Format	Input/output format of the tools and other specification formats
Repository	A URL indicating the repository of the tool or another source where it can be downloaded
Article	A (preferably DOI) link pointing to a publication about a tool
Concept	All tags, application domains, etc. can be concepts. Some specific concepts are created as sub-classes to allow domain experts to modify the ontology file for tool classification
PV	Seven hierarchy levels (PV0–PV6) that classify tools. Each tool receives a single classification
Conference	The conference where an article was published
Person	Persons related to a tool, proceedings or any other concepts
Writer	Sub-class of Person: Authors of an Article
CodeContributor	Sub-class of Person. Contributor to a Repository

**Table 2:** The classes in the ProVerB ontology with an example of the corresponding data for one concrete tool

Class	Example
Tool	JayHorn
Format	Java bytecode (it supports Java class files, Jar archives, or Android apk)
Repository	https://github.com/jayhorn/jayhorn
Article	https://doi.org/10.1007/978-3-030-72013-1_29
	https://doi.org/10.1145/3340672.3341113
	https://doi.org/10.1007/978-3-030-17502-3_16
	https://doi.org/10.1007/978-3-319-41528-4_19
Concept	Java; Modelchecking
PV	**PV4**
Conference	TACAS’21; FTfJP’19; TACAS’19; CAV’16
Writer	Hossein Hojjat; Temesghen Kahsai; Philipp Rümmer; Huascar Sanchez; Martin Schäf; Ali Shamakhi
CodeContributor	MartinSchaefPhilippRuemmerAliShamakhiHuascarSanchezTemesghenKahsai; etc

This process can be automated with GitHub workflows to run once per week and automatically generate a PR with the updates for the data set. The PR can then be reviewed before merging to ensure the data is of sufficient quality.

#### Author-contributor relations

With the previously explained setup we can also collect data about authors of papers and contributors to repositories. Specifically, we can explore “same-as” relationships to identify people who were both author and code contributor. This can provide valuable information about who to contact when someone has questions about a tool.

This data is collected through the APIs, though it is not yet added to the data set or shown on the website. The topic of author contributions, as shown by Corrêa Jr., Silva, da F. Costa, and Amancio [26], is far from being simple and/or resolved even for “normal” academic literature, and deserves even more careful investigation if we take tool making and empirical validation into account.

### Data set statistics

The data set contains 427 tools, 26 specification formats and 71 tags. The tools are split over the PV-levels as follows:

- **PV0**: 16 — cf. Sect. 5.1
- **PV1**: 98 — cf. Sect. 5.2
- **PV2**: 84 — cf. Sect. 5.3
- **PV3**: 74 — cf. Sect. 5.4
- **PV4**: 101 — cf. Sect. 5.5
- **PV5**: 13 — cf. Sect. 5.6
- **PV6**: 13 — cf. Sect. 5.7
- No PV: 46
- To be categorised: 8

**Table 3:** An overview of how many tools were identified in the CAV and TACAS proceedings

	Tools	Prototypes	No tool
CAV	257 (50%)	54 (10%)	95 (22%)
TACAS	94 (18%)	0 (0%)	19 (4%)
Overall	351 (68%)	54 (10%)	114 (22%)

Table 3 gives an overview of how many tools were identified in the CAV and TACAS proceedings respectively. The papers that presented unnamed prototypes were counted separately and excluded from the data set. Papers that did not discuss any implementation, such as theoretical papers or case studies, counted towards the “No tool” column. Overall, 78% of the papers that we looked at included some implementation, 68% of which were identifiable tools and 10% were prototypes. We suspect the percentages to be considerably lower, had we chosen other conferences without a strong tool focus.

The light snowballing principle that we have mentioned above (another tool page is added if at least two existing entries refer to the same tool which is not yet in the data set) led to adding another 76 tools to the data set.

We consider limitations of our data set and the process of creating it, at the very end of the paper, in Sect. 6.1.

#### Data enrichment statistics

400 out of 427 of the markdown files were included in the data enrichment process. The other files were excluded as they were READMEs and pages that did not describe tools but specification formats.

In total, we could automatically retrieve information about 256 of 269 repositories. This information included code contributors, the last commit date and the “About” section shown on a repository’s page. For the articles, we were able to enrich 518 of 525 articles successfully. For each article, it retrieves the title, abstract and authors. Some repositories and articles could not be enriched as this data was not available through Github’s, Springer’s or Crossref’s API. For example, some repositories are hosted on organisation-specific GitLab instances and some articles link to an organisation’s website instead of a DOI.

In total 1419 code contributors and 1188 authors were identified. Of these contributors, 1086 people have provided a name which can be used to identify same-as relationships with authors. In total, 273 same-as relationships have been found when looking for exact name matches. 169 tools contain at least one expert who contributed to both the paper and the code. The actual number is expected to be much higher as we only considered exact matches and not all code contributor’s have provided a name that can be matched to.

## Trends in PV-levels

In this section, we identify different subgroups within each PV-level of the megamodel.

### **PV0**: potential tools


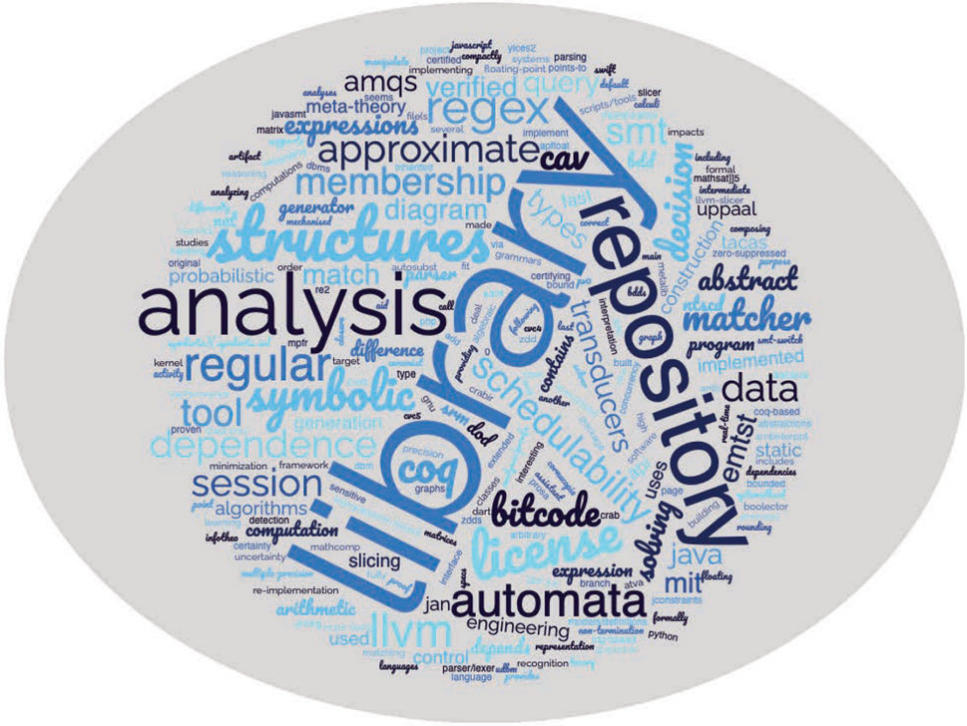


At the time of writing of this paper, ProVerB had 16 tools on **PV0**. It is the absolute minority compared to other categories, which is intentional due to the inherent non-verifying nature of **PV0**. As the word cloud, generated from tool descriptions and visualised above, suggests, it is more about analysis than proving, and more about “matching” than “checking”, and some of these “tools” are mere libraries for larger packages like Coq and Uppaal. 13 of the **PV0** tools provide facilities to work with various kinds of seemingly formal artefacts: grammars, regular expressions, automata, decision diagrams, session types, and floating point numbers. However, there is simply not enough formal rigour in the way these tools operate these artefacts, for us to consider them truly a part of the program verifier’s arsenal. As an example, consider ANTLR [69]: given a grammar, it generates a parser for it. However, it does so without the grammar being perceived, modelled and transformed as a mathematical object. If the user provides ANTLR with a grammar which is unconnected or ambiguous, then the generated parser will be faulty, and no warning might be issued.

Two remaining **PV0** tools are, in fact, repositories: Ceramist [42] and Prosa [43] are libraries that store formal artefacts (definitions and proofs) but by themselves neither provide arguments about their correctness, nor verify those (both rely on Coq). The last **PV0** tool is Smt-Switch [60], a collection of abstract classes that, if inherited from and implemented, can help integrate SMT solvers—again, this library by itself is definitely related to the PV domain, but does not help bring any correctness guarantees.

What all **PV0** tools have in common is their position on the verification diagram we have shown in Sect. 3: they are claims without arguments, without a prover and without a verifier. The claims can be formal, but the surrounding context does not qualify as PV tool support.

### **PV1**: essential tools


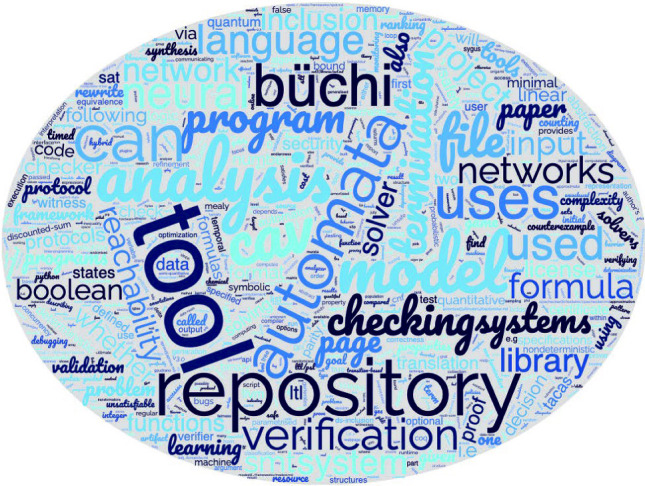


Out of 98 tools on the **PV1** level, 19 can be seen as frameworks enabling their end users to work with certain models/abstractions in a formal way. For instance, Frama-C [24] contains functionality to treat C programs as formal artefacts and thus can be used to build different program analyses on top of it; BINSEC [31] provides similar functionality and opportunities to implement binary level code analysis; there are comparable tools that deal with Büchi automata, symbolic automata, decision diagrams, temporal logic formulae, etc. 9 more tools could be seen as limited frameworks that are developed specifically to compare two models in a formal way. For example, SPAN [13] computes whether two protocols are indistinguishable, and RABIT [1] checks inclusion of languages generated by two Büchi automata. Another 8 tools can be seen as normalisers that bring a given model to some well-defined canonical state: Mealy machines and Büchi automata can be automatically minimised, quantified Boolean formulae can be simplified and turned into dependency quantified Boolean formulae, etc.

21 different **PV1** tools are linters, type checkers and checkers of other kinds of properties that are fixed and hardcoded into the tool (we will see checkers of user-specified properties on **PV3**). Such properties can include conformance, semantic preservation, type safety, automata emptiness, safety of Markov decision processes, thread safety, etc. Reachability and termination analyses, due to their internal workings, we count towards another category, which includes metric calculators and tools that compute a set of possible states of a model or infer ranking functions, or compute upper and lower bounds of something — there are 34 of them in total.

Finally, the remaining 7 tools can execute models, simulate their behaviour, (partly) visualise them and resolve them otherwise: Murxla by Niemetz, Preiner, and Barrett [64] fuzzes SMT solvers, CabPy by Baier et al. [9] solves a two-player reachability game, Oink by van Dijk [85] solves a parity game, jcstress by Shipilëv [79] and PROVER by Ryou et al. [75] execute test cases in a specific order, CLEAR by Barbon, Leroy, and Salaün [11] and dtControl by Ashok et al. [7] visualise the problematic part of a labelled transition system and previously externally synthesised controller code, respectively.

### **PV2**: creational tools

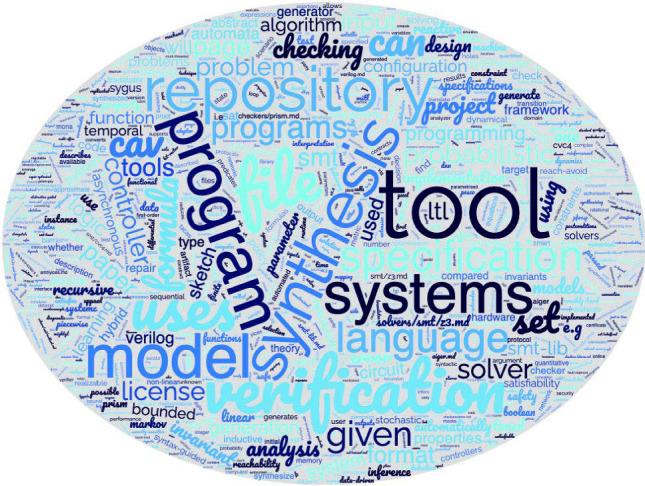



There are 84 tools in **PV2**, their descriptions visualised as above. The largest identifiable group, with 37 members, consists of tools providing correct-by-construction artefacts given a specification: some synthesise a controller from an LTL formula, others generate a dynamic neural network for a given grid, some generate tests for a given circuit, while others specifically generate classes that attempt to violate given properties. This group of tools can produce fairly formal artefacts that are automatically verifiable, but they do not provide any verifier means themselves. 8 more tools perform limited versions of the same process, generating only enough content to fill in holes in an already partially existing model or program. For instance, $\tau$-DIGITS by Drews, Albarghouthi, and D’Antoni [33] fills holes in a given loop-free program from a probabilistic specification of its desired behaviour, and MOVEC by Chen, Wang, Zhu, Xi, and Yang [25] performs aspect weaving. Two more tools (DIGITS by Albarghouthi, D’fAntoni, and Drews [2] and TarTar by ölbl, Leue, and Wies [53]) specifically propose repairs as code fragments meant to substitute existing code fragments assumed to be faulty.

The second popular group contains 19 tools that encode or transform the artefact from one format or formalism to another. This group covers tools for sequentialising parallel C code (MU-CSeq by Tomasco et al. [82]), or transforming irreversible programs into reversible circuits (ReVerC by Amy, Roetteler, and Svore [4]). There are several tools on this level that operate on temporal logic formulae, making a timed automaton (MightyL by Brihaye, Geeraerts, Ho, and Monmege [21]) or an Electrum model (Cervino by Peyras, Bodeveix, Brunel, and Chemouil [70]) or another temporal logic formula in a different dialect (MLTLconverter by Li, Vardi, and Rozier [59]) from them.

8 tools can be used to refine specifications: for instance, by inferring type annotations from an untyped program such as Typpete by Hassan, Urban, Eilers, and Müller [46], or generating permission pre- and postconditions for Viper programs like Sample by Dohrau, Summers, Urban, Münger, and Müller [32] does.

Finally, 10 tools generate configurations or settings for other tools, such as PeSCo by Richter and Wehrheim [73] which generates the best fitting configuration for CPAchecker by Beyer and Keremoglu [17] that fits previous experiences; or SATzilla by Xu, Hutter, Hoos, and Leyton- Brown [92] that decides which solver to call per instance based on predictors.

### **PV3**: property checking tools

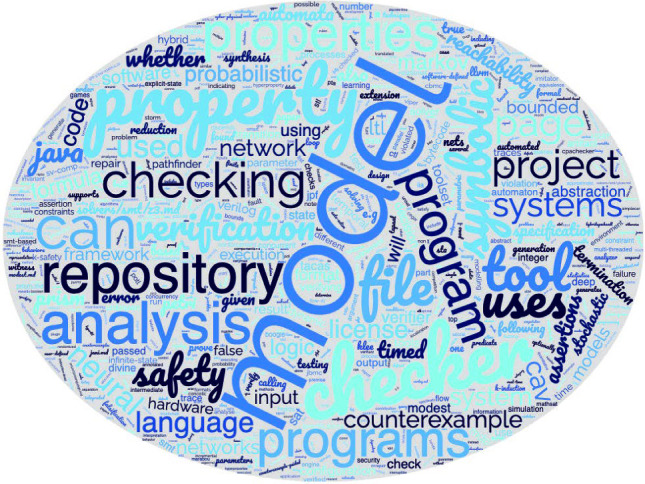



**PV3** currently has 74 tools. Within **PV3** we can clearly identify three main subgroups: property checkers, assertion checkers and program repair tools.

The first group consists of 40 tools that check properties for some form of model such as automata or network models. For instance, STAMINA by Neupane, Myers, Madsen, Zheng, and Zhang [63] can be used to check properties of infinite-state continuous-time Markov chains.

The second group consists of 28 tools that check assertions for concrete artefacts. For example, SecC by Ernst and Murray [34] can check information flow properties, expressed as assertions, for C programs.

Four tools: Forester by Holík et al. [48], SymDIVINE by Mrázek, Bauch, Lauko, and Barnat [62], Trainify by Jin, Tian, Zhi, Wen, and Zhang [50] and VeryMax by Borralleras et al. [20] — fall in between these two groups. The first two of these work on LTL formulae as properties, but apply them on real C/C++ code (SymDIVINE allows both “normal” assertions and LTL formulae). Trainify checks ACTL properties for Deep Reinforcement Learning systems defined in Python. VeryMax works both on programs (C/C++) and models (transition systems).

Finally, there is also a small but growing group of tools that focuses on program repair: AllRepair by Rothenberg and Grumberg [74] and NNRepair by Usman, Gopinath, Sun, Noller, and Păsăreanu [84]. These tools both identify faults in the program, like other tools in **PV3**, and they also propose a way to fix it.

### **PV4**: specification checking tools


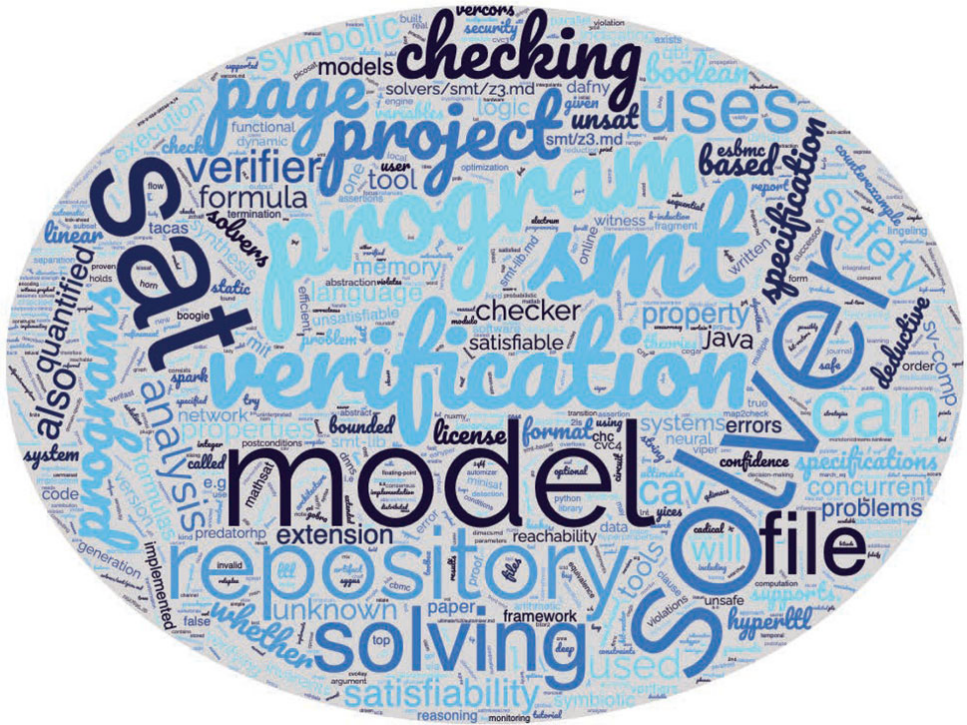


Currently, **PV4** is the largest category with 101 tools, the descriptions of all of them also used for a word cloud above. The largest group (51) of tools within **PV4** are the solvers. These tools produce a satisfiability result for SAT (satisfiability), SMT (satisfiability modulo theories), QBF (quantified Boolean formulae) or CHC (constrained Horn clauses) problems. Because these tools verify a specific property (namely, satisfiability), one may have expected to find them in **PV1**. However, these tools typically generate interpretations for the given problem to show that it is (un)satisfiable. So, internally each of these tools consists of two essential parts: the **property generator** which generates the interpretation and the **verifier** which checks whether this interpretation makes the formula satisfiable. This group also contains the tool that is referred to the most often in our data set — namely, Z3 by Moura and Bjørner [30]. It belongs to SMT solvers together with 17 other tools; there are also 22 SAT solvers; 3 CHC solvers; and 6 solvers of other kinds.

Many PV tools from other levels encode their problems into satisfiability problems and then use one of the tools in this group as a back end.

**PV4** also includes 25 tools that generate properties or check built-in specifications typically depending on the domain that the tool targets. Some examples of built-in specifications that are checked, include memory safety, data-race freedom, termination and absence of runtime errors. Many of these tools also provide support to check user-written properties. For instance, Gobra by Wolf et al. [90] can check user-written assertions for Go programs as well as memory safety, data-race freedom and crash safety.

Finally, there is a small group of what we can call language workbenches [39], and we strongly suspect that there are more of this kind that escaped our selection only because nobody published about them directly at CAV and TACAS recently. A language workbench was envisioned in 2005 as a set of tools aiding the language engineer to design, implement and integrate a collection of domain-specific languages into one unified solution. Some of the popular language workbenches in model-driven software engineering include Xtext, MPS, MetaEdit+, Rascal and Spoofax. The two language workbenches that we have found mentioned for the domain of program verification, were DLC by Evrard [35], which can automatically generate distributed implementation of concurrent systems modelled in the LNT language, which can be verified using the CADP toolbox; and PrDK by Arbab [51], a development kit for programming communication protocols.

### **PV5**: fully controlled verification tools

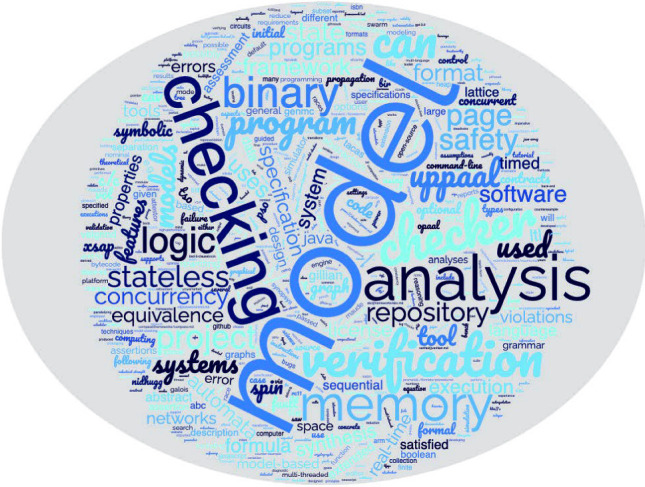



Continuing the same trend, on **PV5** we see a uniform group of 13 verification workbenches. These are tools that allow users to write their own specifications and combine these together with desired properties into a formal mathematical representation. These formal representations can then be compared with representations of programs or their properties for the verification. Users can have very fine-grained influence on the results of these tools because they are allowed to write their own specification. For example, Attestor by Arndt, Jansen, Katoen, Matheja, and Noll [6] allows the user to specify the initial heap configuration and the behaviour of the garbage collector that should be taken into account when verifying a property for a Java program. Similarly, UPPAAL by Bengtsson, Larsen, Larsson, Pettersson, and Yi [14] is a workbench for automatic verification of safety and bounded liveness properties of real-time systems modelled as networks of timed automata.

### **PV6**: proving tools

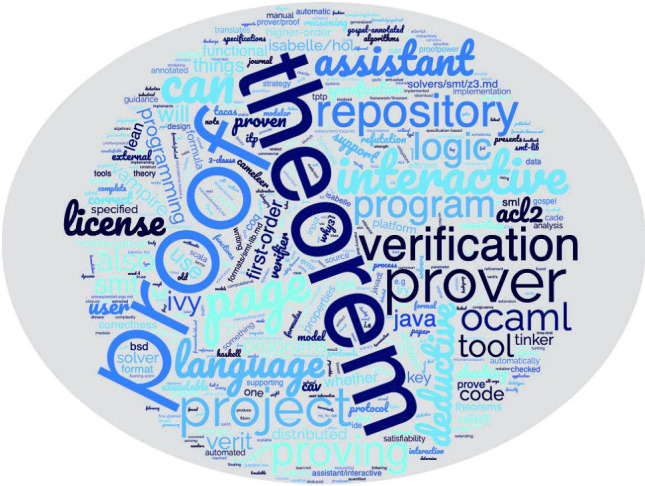



All 13 tools in **PV6** are proof workbenches. Many of the tools in previous categories give a yes/no answer to indicate whether a property holds, and in any case allowing at most some influence on the property generating and handling process, but not on the final proof. **PV6** tools, however, will help the user to construct and infer the correctness of a proof that shows **why** a property is true or false. Some well-known tools in **PV6** are Coq by Bertot and Castéran [15], Isabelle/HOL by Nipkow, Wenzel, and Paulson [65], Lean by L. de Moura and Ullrich [29] and Vampire by Kovács and Voronkov [54]. Their comparison is a highly nontrivial task even for professional mathematicians [87].

## Conclusion & roadmap

Our contribution of this paper is two-sided. On one side, we have analysed a fairly complex domain and turned one of the commonly used visualisations of its core processes into a full fledged megamodel that helped us to split the domain into much more intelligible smaller categories. On the other side, we have processed hundreds of academic papers published across several recent years, classified them according to the proposed megamodel and generated a user-friendly website allowing software engineers to compare and assess tools in a bit more secure, complete and safe way than before.

The megamodel that we have presented, identifies the different type of program verification tools that we found existing or that can possibly be made to exist. This megamodel is based on the classic division of roles in a correctness proof as introduced by Goldwasser et al. [41] that is currently accepted by the computational community. Our megamodel divides the different types of tools into seven categories: **PV0**, **PV1**, **PV2**, **PV3**, **PV4**, **PV5** and **PV6**. These categories are increasingly more demanding and increasingly more powerful: it is possible to gain some benefit from a **PV0** or **PV1** tool within the first day of being introduced to it, but much further refinement and improvement might not be possible; on the other side of the spectrum, **PV6** tools can do almost everything, and require a relevant PhD degree to operate. Thus, there is no discussion on “what is the best PV-level”, just a classification that helps to match a tool to customer needs.

To bring the megamodel to life, we have designed a metamodel to hold semi-structured information about a PV tool, including its PV-level, name, input/output, etc, and instantiated it for all tools that we have found being mentioned and used in the last five years of two top conferences in the field: CAV and TACAS. Our data set at the time of writing consists of 450+ tools, formats and libraries. By setting up a megamodel as well as a data set, we hope to provide both a theoretical as well as a practical starting point to get into the world of PV tools and methods. A good starting point for browsing and exploring ProVerB would be its hypertext frontend: http://slebok.github.io/proverb/ which also contains links to other sites (GitHub, DOIs, etc) for each tool.

### Threats to validity

*Conclusion validity.* All the PV-classifications that we have performed, come from our personal interpretations of the contents of a fairly large body of fairly complex academic papers. Thus, it is possible that some tools have been misclassified as belonging to one level while they actually belong to another level. To prevent misclassifications, the authors were actively double checking each other’s verdicts and had extensive discussions about arguable conclusions. Eventually we plan to reach out to authors of all tools included in ProVerB individually, with a detailed explanation of the seven PV-levels and a request to review our summaries and refine them, possibly leading to reclassifications. If such a community effort causes a noticeable resonance, it would be possible to eliminate this threat entirely.

*Internal validity.* Since our project is more of an observational and classificational nature, initially we did not attempt to establish any causal relationships. Hence, internal validity was not among our major concerns. However, since the moment we started enhancing the data set with other sources of linked data such as GitHub and SpringerLink API, it becomes increasingly more relevant to correctly establish contributor identity equivalence across multiple platforms with varying usernames and non-strictly matching names [38].

*Construct validity.* As we have explained in Sect. 3, our megamodel was designed based on the classic division of roles in a correctness establishing setup as described by Goldwasser et al. [41], in the modern reinterpretation by Wigderson [88]. By reusing a model that originates from the right domain, we hope to have found a mature foundation that will allow us to classify any possible tool in the future by matching its components and concepts to the claim, the prover, the arguments and the verifier. Only if we encounter future tools that do not fit into this model, will we have to redesign the megamodel again. However, in the works of Wigderson [88], generalising the notion of a proof from being a unidirectional communication from the prover towards the verifier, to a bidirectional series of communications, handling interactivity, errors, randomness and other natural aspects of computation, has opened a lot of doors and led to the discovery of a number of complexity classes with a distinctly higher expressive power. For instance, relying on more than two verifiers at the same time is not uncommon in PV, but this is mostly done for practical considerations such as trying all available ones to watch only the fastest complete its proof. It is neither considered nor suspected that multi-prover or multi-oracle PV tools can lead us to a broader computational class. Since this has not been researched or established before, we also do not consider such multi-tier setups as one of the PV-levels explicitly.

*External validity.* We have gathered data from publications at CAV and TACAS, which seemed like a good choice of information since both favour papers about program verification tools to non-tool papers and non-PV content. However, there are more venues that target the program verification field (POPL, PLDI, FASE, LICS, etc). It is unknown at the moment what biases we have created in the data set by limiting ourselves to only CAV and TACAS and related papers, techniques and tools. While limited, the number of tools (380+) included is significant, and they seem reasonably spread out among the different PV-levels. We see that the most popular tools are included, partly because we also include tools if they are referred to by at least two other tools. So, while perhaps limited on the grand scheme of things, we think that this is a good starting point for the data set.

### Roadmap for the future

With the megamodel designed, and the initial data set collected, our main focus for the future is the usage of our work. The PV-levels naturally cover the entire spectrum of all possible tools from informal/semiformal to self-validating, so the usual future work claim of adding more levels to the megamodel, cannot possibly apply here. Thus, below we discuss several concrete scenarios to show how it can be used and extended to cater for those uses.

*Scenario 1:* the first scenario, which closely resembles our original goal of collecting and classifying available program verification tools for ourselves, is to *help users find suitable tools*. Even now potential users of any of the tools listed at the ProVerB website, can explore the data set in different ways, such as systematically covering some PV-level after determining which one is needed, or browsing through tools listed under one of the tags (e.g. “LLVM” or “Smart contract”). To improve the usability in this scenario, we believe it would be useful to develop a decision tree to help people find the right tool. Such a decision tree can take the client’s requirements into account such as the domain to which it is applied and the problems it should solve.

One of the aspects of seeking the right tools for the job that we have not considered before, is tool popularity among other potential users — for some definition of it. However, there are many ways to assess popularity or utilisation of program verification tools, such as:

- **Presence:** in our study we have declared that even one use or mention of a tool justifies its addition to ProVerB — however, more frequent appearances at conferences and workshops, as well as prominence in tutorials and keynotes, may indicate higher popularity. In some subdomains of program verification contests are being held regularly, and if a tool appears there often, it means it is highly competitive among cutting edge alternatives.
- **Citations:** the number of papers referred to either the main paper introducing the tool in question, or collectively to all papers written about the tool. This can be possibly calibrated by tracking the age of citations as well, to avoid the bias towards older tools.
- **Usage:** many platforms allow tool makers to track the number of downloads, installations or active users. Tools mature enough to be used in the industry can also often be tracked with respect to their adoption, either directly through companies applying them to solve their problems, or indirectly by counting published case studies and success stories.
- **Community:** engagement can be a good proxy for a tool popularity, hence the reliance in many studies on counters like GitHub stars and forks, or the number of contributors, issue resolutions or commits.
- **Social Media:** presence on social media platforms goes beyond simple vanity and can also mean that blogs/podcasts, discussion fora/groups, Discord/Slack servers, Reddit/StackOverflow sections, etc, are readily available to support newcomers learning to use this tool.
- **Surveys:** tool popularity can be measured directly by asking a significant number of researchers and practitioners to express their preferences by filling in questionnaires. This is a labour intensive initiative but yielding qualitative insights into actual tool popularity and perceived effectiveness.

There are also composite methods of expression popularity and adoption of a piece of software, such as Software Universe Graphs by Kula, De Roover, German, Ishio, and Inoue [56]. In any case, we note that popularity has been found to not correlate that much with other aspects of software quality such as defect density [76] or security [80]. Other studies have even found an inverse correlation, such as Alsmadi and Alazzam [3] who observed that projects with higher number of downloads also tend to have higher cyclomatic complexity.

*Scenario 2:* the second scenario is similar to the first one, but the selection is done in the presence of a *knowledgeable consultant*. Such an expert has sufficient knowledge about the field of program verification and thus can determine what type of tool suits the client’s problem. Exploring the domain together with the client could potentially lead not only to reaching its intended goal of finding and selecting the right tool, but also to insights in requirements elicitation. It is well-known that exploring the solution space often leads to discoveries on the problem definition side. For this scenario, the most useful improvement would be to subdivide the PV-levels further. At the moment the levels are neither uniform nor equidimensional: some are naturally larger, others contain substantially sized identifiable subcategories.

Different aspects can be of more importance in this scenario, or at least can gain more weight due to the presence of a verification expert acting as a requirements elicitor, such as:

- **Performance:** efficiency of the candidate tool can be assessed by either their known position in the last relevant competition, or by observing how consistently it passes appropriate applicable benchmarks.
- **Flexibility:** adapting the tool to the needs of the user might involve scalability issues, assumptions about input data, customisability and extensibility, as well as some other similar details that can subtly influence the choice.
- **Integration** with widely used formats, platforms, frameworks or toolchains can enhance the tool’s chances of being a part of the ultimate solution.
- **Learnability:** some tools are known to be more user-friendly than others, or having a particularly steep or gentle learning curve. This can be supported by availability and reliability of documentation, by accessible tutorials and books, as well as by other means of skill transfer.
- **Community** can play a role just like in the case of popularity above. Ongoing support and continuing improvement weighs just as much here as having an active group of core developers and helpful and inclusive learning community.
- **Risks**, if known, can be weighed realistically and mitigated by planning for contingencies. This applies to future plans for dealing with the tool becoming obsolete, relying on older dependencies, keeping vulnerabilities unresolved, etc.

*Scenario 3:* another usage scenario focuses on using the available data, and a similar format, to *improve artefact evaluations or paper reviewing* process. In artefact evaluations, reviewers can typically indicate what artefacts they would be interested in reviewing based on a title and/or abstract [55]. However, these do not always provide a clear description of the tool’s capabilities due to which a reviewer end up having to review a tool outside of their field of expertise. Using a format as in our data set would provide a more structured approach to describe the tool and can be helpful to correctly identify what tools’ capabilities are to prevent such situations. Moreover, if this format can be incorporated into artefact evaluations as a required part of the submission, this provides a distributed approach that allows us to keep the data more easily up to date.

Other key aspects that may prove useful in the context of this scenario, are:

- **Evaluation Criteria** can be standardised and applied consistently across different tools, improving the rigour and effectiveness of the entire artefact evaluation process. It might even be possible to devise a standardised methodology to evaluate tools according to the PV-level they find themselves on.
- **Submission Guidelines** are becoming clearer each year, but perhaps clear positioning of a submitted tool on a PV-level and clearly stating its input and output formats, as well as filling out other fields that we ended up using within ProVerB, could align tool authors’ and evaluators’ expectations better.
- **Tool-Agnostic Benchmarks** can be based on data sets that are specific to a PV-level or to a format or to a combination of a tags/concepts. This will not only improve replicability, but also encourage and significantly simplify cross-tool comparable studies.
- **Automation of Evaluation** is the ultimate goal of artefact assessment. While there is perhaps still place for human evaluation as a part of it, just like there is a place for it for peer reviewing papers, many steps such as conformance to a declared format or the ability to produce the same outputs as reported in the accompanying paper, can and should be automated to avoid manual labour and human bias.

*Scenario 4:* for all three of the scenarios described above, it can be useful to further extend our data set. This can focus on either adding new entries to the current data set, or to extend the information that is available for the current tools. At the moment, in order to classify a new tool that is absent from the data set, into an appropriate PV-level, one would basically need to read this paper and combine their understanding of what the tool does with their understanding of the what each PV-level stands for. It will be more appropriate to design a specific decision tree with clear questions, answers to which will unequivocally lead to one level or the other. In a way, this is an “implementation detail” since it might not require deeper understanding of the megamodel, but it is also known that explicitly *renarrating* a megamodel significantly increases its appreciation among domain experts and other users [93].

Pragmatically, contributing to the ProVerB repository is straightforward: the data set is a GitHub repo which can be forked and worked on, with a pull request asking to merge it back to the original. In order to add a new tool, an external contributor can create a new Markdown document from the template — manually or with one of the available scripts (e.g. parsing a proceedings volume and suggesting a number of tools found there heuristically). All the refinement features described above, also remain accessible: for instance, it is sufficient to add DOIs of papers and URIs of source code in order for our GitHub workflow to pick them up and replace bare links with paper titles and to add last activity information from accessing the APIs.

Additionally, it is interesting to investigate the possibility of combining our ontology with others, including, e.g. the runtime verification tool taxonomy by Falcone et al. [36] and the repository structure proposed by Schlick et al. [77]. When extending the ontology, it is important to consider how to incorporate grey literature. So far, we have only included tools that were introduced in the academic literature. However, David et al. [27] have shown that, for modelling tools that support blended modelling, grey literature had a higher ratio of tools introduced per literature source. In the grey literature, they identified 68 tools in 1494 sources whereas, in the academic literature, they identified 68 tools in 4975 entries. It is unclear whether such a ratio would be similar in the field of program verification, a field known for its theoretical difficulties. Nonetheless, in all likelihood, we are currently missing some tools that have only been discussed in grey literature.

## Footnotes

1 The data set has also been archived at https://doi.org/10.4121/20347950

2 CAV: http://portal.core.edu.au/conf-ranks/331/

3 TACAS: http://portal.core.edu.au/conf-ranks/1818/
